# Is inhibition of kinase activity the only therapeutic strategy for *LRRK2*-associated Parkinson's disease?

**DOI:** 10.1186/1741-7015-10-20

**Published:** 2012-02-23

**Authors:** Iakov N Rudenko, Ruth Chia, Mark R Cookson

**Affiliations:** 1 Cell Biology and Gene Expression Section, Laboratory of Neurogenetics, National Institute on Aging, National Institutes of Health, 35 Convent Drive, Room 1A-116, Bethesda, MD 20892-3707, USA

## Abstract

Mutations in the leucine-rich repeat kinase 2 (*LRRK2*) gene are a common cause of familial Parkinson's disease (PD). Variation around the *LRRK2 *locus also contributes to the risk of sporadic PD. The LRRK2 protein contains a central catalytic region, and pathogenic mutations cluster in the Ras of complex protein C terminus of Ras of complex protein (mutations N1437H, R1441G/C and Y1699C) and kinase (G2019S and I2020T) domains. Much attention has been focused on the kinase domain, because kinase-dead versions of mutant LRRK2 are less toxic than kinase-active versions of the same proteins. Furthermore, kinase inhibitors may be able to mimic this effect in mouse models, although the currently tested inhibitors are not completely specific. In this review, we discuss the recent progress in the development of specific LRRK2 kinase inhibitors. We also discuss non-kinase-based therapeutic strategies for LRRK2-associated PD as it is possible that different approaches may be needed for different mutations.

## Introduction

Parkinson's disease (PD) is a relatively common age-related neurodegenerative disorder [1]. In the past few years, it has become recognized that there are single-gene disorders that are clinically and pathologically similar to sporadic PD [2]. Furthermore, a series of genome-wide association studies (GWAS) have identified a number of loci that contribute to the risk for sporadic PD [3-8].

The most common single genetic cause of autosomal dominant PD is missense mutations in the leucine-rich repeat kinase 2 (*LRRK2*) gene [9,10]. Variation around the *LRRK2 *locus also appears to be a risk factor for sporadic PD [4,11]. Many single-nucleotide substitutions have been reported in the *LRRK2 *gene, but only six have been verified to be pathogenic [12,13]. These mutations are located predominantly in the enzymatic domains of LRRK2, including the kinase domain for which LRRK2 is named. There is a particularly common mutation, G2019S, in the kinase domain that increases the kinase activity of the protein (reviewed in [14]).

For many LRRK2 cases, the clinical signs in affected carriers of the mutation are remarkably similar to idiopathic PD and include tremor, rigidity, postural instability and bradykinesia [15-17]. There is some variation, as individual patients may have developed amyotrophy [18], whereas in one of the first described families, there is more frequent gait disturbance and less frequent tremor compared to sporadic PD [19]. However, the general picture is that LRRK2 causes a disease that overlaps substantially with sporadic PD.

Because LRRK2 is a kinase, it has been suggested that targeting kinase activity might be a therapeutic strategy for familial PD. Furthermore, because LRRK2-related PD and sporadic PD are clinically similar, it might be extrapolated that targeting kinase activity might also be helpful in treating the more common idiopathic form of the disease.

In this review, we discuss the evidence underlying the idea that LRRK2's kinase activity might be modified to protect against PD. We also focus on the alternative idea that there are other aspects of LRRK2 function that might equally be addressed therapeutically. To understand these two ideas, it is important first to identify the mutations in LRRK2 that are associated with PD and how these affect protein function.

## Pathogenic mutations in PD affect protein function

LRRK2 has a complex multidomain structure. The central part of the protein contains three independent domains. The Ras of complex protein (ROC) and C terminus of ROC (COR) bidomain is characteristic of the ROCO family of proteins [20]. The ROC domain binds GTP, and the COR domain is thought to be a regulator of ROC GTPase activity [21]. LRRK2 is an active protein kinase [22,23], although the true physiological target of this kinase activity has not been resolved.

It has been proposed that the ROC-COR and kinase domains of LRRK2 may control each other. Mutations in the ROC domain that prevent GTP binding are kinase-inactive, leading to the suggestion that the GTPase activity regulates kinase activity (reviewed in [14]). Recent evidence presented by two independent groups, however, showed that kinase activity of LRRK2 is not increased by adding GTP as would be predicted with the use of this model [20,24]. An alternative interpretation is that the GTP-binding mutants disrupt or destabilize the ROC domain, resulting in a loss of kinase activity as a secondary output. It is possible that instead the kinase domain of LRRK2 regulates the ROC domain and thus that the kinase domain is the modulator of activity rather than the output of the protein [12,25]. However, whether this regulation actually happens *in vivo *is unknown [14]. Currently, it is thought that there is some form of regulation between the enzymatic domains of LRRK2, although the details of the mechanisms still need to be resolved.

The flanking ankyrin/heat repeats and leucine-rich repeat (LRR) domains at the N terminus and WD40 domain at the C terminus are thought to participate in protein-protein interactions. However, the physiological role or identification of interactors of these domains and how these domains dictate LRRK2 function remain unclear. There may be a role for some of these regions in the control of LRRK2 kinase activity, as the removal of sequences from the C terminus has been shown to abolish the phosphorylation ability of LRRK2 [26,27]. Overall, the available data suggest that LRRK2 is a protein that has kinase activity, binds GTP and could play a scaffolding role via its protein-protein interaction domains.

Several groups have shown that the G2091S mutation augments *in vitro *kinase activity of LRRK2 by two- to fourfold [22,23,26,28-35]. This might suggest that all mutants have a simple gain of kinase function mechanism. I2020T is located at the next residue of LRRK2 as G2019S, but it has either a modest increase [36,37] or a reduced [26] or no effect [35] on kinase activity levels compared to wild type (WT). The simplest interpretation is that the I2020T mutation does not increase kinase activity, at least in the way it is currently assayed, which contrasts with G2019S. Therefore, whether all mutations, even those in the kinase domain, increase the kinase activity of LRRK2 is uncertain. Some variants seem to consistently decrease kinase activity, including the G2385R variant [26] that is a genetic risk factor for PD in some populations. Therefore, these variants would seem to be pathogenic by a non-kinase-dependent mechanism.

Another group of LRRK2 mutations is clustered in the ROC-COR bidomain. The R1441 position in the ROC/GTPase is mutated to cysteine, glycine or histidine (that is, R1441C/G/H) in families with PD [10,38-42]. Several families who have a Y1699C mutation in the adjacent COR domain have been described [10,43]. A recent addition to this mutation cluster is N1437H, which was found in a large Norwegian family with autosomal dominant parkinsonism [13]. Overall, the balance of the evidence is that R1441 and Y1699 mutations raise the kinase activity of LRRK2 only very modestly. In contrast, the R1441C/G [44-46] and Y1699C mutations consistently lower GTPase activity [46]. The GTPase activity for N1437H has not been determined, but it has been shown that this mutant has increased GTP binding [13], which is consistent with lower GTPase activity. The reported GTP-binding capacity of R1441C/G and Y1699C is less consistent, with some reports of an increase and others finding no change in GTP binding [45,47,48]. These effects are probably related, as it has been proposed that the ROC and COR domains may interact with each other. R144C destabilizes the ROC domain [49], whereas Y1699C substitution strengthens the intramolecular ROC-COR interaction [46]. Although the precise details still need to be worked out, this suggests that interactions in the ROC-COR bidomain are important in controlling the GTPase activity of LRRK2 and that this may be dysregulated by mutation.

Although the molecular mechanisms by which mutations affect LRRK2 seem to differ, there are phenotypes reported at the cellular level that appear to be consistent between mutations. At least under the conditions of high-level expression of LRRK2 *in vitro*, mutant forms of LRRK2 are acutely toxic [23,27,29,47,50]. For G2019S LRRK2, there is evidence that cell death also occurs *in vivo *with viral vector-mediated expression [51,52].

In cultured neurons, neurite shortening is also a rather consistent phenotype associated with mutant LRRK2, even in models where cell death is not apparent [28,53-58]. Importantly, all mutations tested to date have one or both of these effects in cells, suggesting that these are common outputs of the same signaling pathways that are affected by mutant LRRK2.

Although not all mutations the have been found to increase kinase activity of LRRK2 using assays that have been tried to date, kinase-inactive versions of the same mutations are not toxic [23,29,55]. Furthermore, a kinase-dead G2019S is not toxic to dopaminergic (DA) neurons *in vivo *[51]. This implies that pathogenesis is dependent on having functional kinase activity. This has led to the idea that the enzyme activity of LRRK2 might be targeted in developing a new therapeutic drug for patients with PD.

## Therapeutic strategies for LRRK2-associated Parkinson's disease

**Figure 1:**
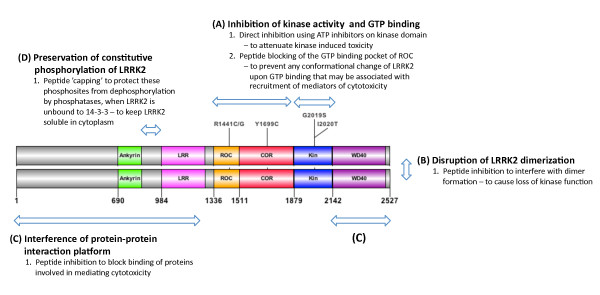
**Schematic representation of potential therapeutic strategies for LRRK2-associated Parkinson's disease**. This diagram highlights some therapeutic possibilities for leucine-rich repeat kinase 2 (LRRK2)-induced cytotoxicity, taking into account how different mutations cause different protein biochemical alterations, recruitment of mediators of cytotoxicity or loss of interaction with regulatory proteins such as 14-3-3. LRRK2 is represented as a simplified linear dimer with the enzymatic ROC-COR-kinase domains, and protein-protein interaction domains, N-terminal ankyrin, LRR, leucine-rich repeat and WD40. Locations of LRRK2 pathogenic mutations are also shown (N1437H, R1441C/G, Y1699C, G2019S and I2020T). Broadly, the possible therapeutic points of intervention for LRRK2 are through **(A) **inhibition of kinase activity and GTP binding, **(B) **disruption of LRRK2 dimerization, **(C) **interference with the protein-protein interaction platform and **(D) **preservation of constitutive phosphorylation of LRRK2. Detailed discussion of these approaches can be found in the main text under **Therapeutic strategies for LRRK2-associated Parkinson's disease**.

The facts that G2019S, the most frequent mutation in LRRK2-associated PD, causes augmentation of LRRK2 kinase activity and that cytotoxicity is dependent on kinase activity lead to the suggestion that inhibition of LRRK2 kinase activity might be therapeutically beneficial. Given the complexity of the LRRK2 molecule, however, it is also possible that there are other aspects of function that might be targeted. Herein we discuss the possibilities of LRRK2 therapeutic agents from the standpoint of kinase inhibition, GTP binding inhibition and other possible alternative avenues for LRRK2-associated PD treatment (Figure 1).

### Targeting LRRK2 kinase and GTPase activity

One approach to manipulating LRRK2 is to develop kinase inhibitors, and this is usually accomplished by making compounds that compete for ATP in the ATP-binding pocket of LRRK2. The selectivity of most kinase inhibitors is therefore incomplete, as most will inhibit other kinases depending on the amount of structural similarity in this region of the kinase. As might be expected, several known kinase inhibitors also inhibit LRRK2, but all have some nonspecific effects [51,59-61]. Because nonspecific inhibition of other kinases can lead to ambiguous results, the ideal compound with which to examine LRRK2 function and dysfunction would have maximum selectivity. Two recently reported compounds, LRRK2-IN1 and CZC-25146, have better selectivity than other kinases for LRRK2 [62,63].

These tools are being used in the laboratory to probe LRRK2 function because they are cell-permeable, but study of them probably will not proceed to clinical trials as they do not accumulate in the brain. Experimentally, one can infuse inhibitors through the cerebral ventricles [51], but this may not be helpful for humans. Therefore, one of the key developments needed in the field to test whether kinase inhibition could work in LRRK2 patients is to develop specific and potent LRRK2 inhibitors, preferably ones that can be given orally (discussed in ***From bench to bedside: clinical trials and future prospects***).

However, there is some data that suggests that chemical inhibition of LRRK2 may not give the same results as expressing a genetically modified kinase-dead protein. Application of kinase inhibitors to cells expressing LRRK2 causes loss of phosphorylation at S910 and S935, located at the N terminus of LRR. This is further associated with the loss of binding to 14-3-3 protein and rearrangement of LRRK2 into filamentous structures. In contrast, kinase-dead LRRK2 retains phosphorylation and 14-3-3 binding and remains cytoplasmic [35]. Therefore, at least in these cellular assays, kinase-dead and kinase-inhibited LRRK2 are not the same.

Worryingly, some pathogenic LRRK2 mutants (R1441C/G, Y1699C and I2020T, but not G2019S) abolished 14-3-3 binding and loss of LRRK2 constitutive phosphorylation. Additionally, these mutants have more LRRK2 inclusion bodies formed in the cytoplasm compared to WT LRRK2 bound to 14-3-3 [35]. Therefore, one must ask if kinase inhibitors might push LRRK2 toward rather than away from a pathogenic form. If this is true, we cannot yet predict the effects of kinase inhibitors and whether these would be the same as those of kinase-dead LRRK2. Although there are data supporting kinase inhibitors preventing neuronal toxicity [51,60], which might more logically be a predictor of therapeutic benefit in patients, experiments performed to date have been done with the less selective agents. Therefore, the data need to be reassessed with more potent and selective kinase inhibitors.

It therefore seems appropriate to think about other approaches for LRRK2 other than kinase inhibition using competitive ATP inhibitors. Kinases often participate in ordered signaling cascades, and thus upstream and downstream effectors of LRRK2 are also attractive targets for pharmacological therapy for patients with PD. Modulating the activity of proteins involved in these cascades could compensate for the impairment of LRRK2 function caused by mutations. For example, as shown in LRRK2 *Drosophila *models, co-overexpression of 4E-BP on the G2019S-mutant background prevented neurotoxicity [30]. 4E-BP is a negative regulator involved in the regulation of translation via binding of eukaryotic initiation factor 4E (eIF4E). Interestingly, mutations in EIF4G1 which bind to eIF4E are also associated with dominantly inherited parkinsonism [64]. Phosphorylation of 4E-BP by LRRK2 causes release of eIF4E from being bound to 4E-BP. Because 4E-BP is also implicated in the mammalian target of rapamycin pathway, it may be possible to regulate the LRRK2-induced effects of 4E-BP by using rapamycin. Tain *et al*. showed this to be a plausible approach in *Drosophila *models, in which they showed that treatment with rapamycin prevented DA neuronal loss [65]. However, the effect of LRRK2 on 4E-BP has yet to be demonstrated in LRRK2 mammalian models.

One other possible target is modulation of LRRK2 GTPase activity. Binding of GTP to LRRK2 could change its conformation, which in turn could mediate interaction with different proteins. GTPases usually cycle between GDP- and GTP-bound states with different affinities for heterologous interactors. For LRRK2, the data on R1441C and Y1699C having lower GTPase activity suggest that the GTP-bound form is associated with disease. Thus blocking the GTP-binding pocket of ROC or stimulation of GTPase activity to limit a pathogenic interaction could be a potential therapeutic target for LRRK2-associated PD [14]. These might be particularly helpful for R1441- and Y1699-mutant LRRK2, but to date no such tools are available that could achieve this goal.

### Strategies based outside the enzymatic regions

Because LRRK2 has many protein-protein interaction domains with no catalytic activity, it is possible that LRRK2 is a scaffold, serving as a platform on which different proteins assemble to perform a specific function. These domains may play a role in mediating the cytotoxicity induced by LRRK2 kinase activity. It has been shown that deletion of N-terminal LRR portion or WD40 of G2019S and/or R1441C rescued LRRK2-induced toxicity in neuronal cells [27,50]. This may be a point of therapeutic intervention by means of peptide inhibition in blocking the binding of proteins involved in mediating LRRK2-induced cytotoxicity as has been done for other globular proteins that interact [66].

Because the kinase activity of LRRK2 is dependent on its dimerization, a way to inhibit kinase activity of LRRK2 selectively could be to disrupt dimeric LRRK2. This could be achieved by using inhibitory peptides to interfere with protein folding or dimer formation. This has been demonstrated to be a successful approach in HIV studies in which an inhibitory peptide was used to inhibit the folding of the HIV-1 protease, an important enzyme in the viral life cycle, thereby inhibiting the life cycle of the virus and controlling AIDS [67]. This therapeutic strategy could be useful for the Y1699C LRRK2 mutant, which has abnormally increased strength of interaction between the ROC and COR domains.

As discussed in ***Targeting LRRK2 kinase and GTPase activity***, some mutant forms of LRRK2 lose the ability to bind 14-3-3 proteins. The mechanisms underlying the loss of constitutive phosphorylation and 14-3-3 binding are not known, but it is possible that the functional effect of these changes is to make LRRK2 less soluble. From this point of view, a possible intervention is to design a peptide that could serve as a "cap" in protecting these sites, when unbound to 14-3-3, from being dephosphorylated by phosphatases. This might more accurately mimic what happens with kinase-dead LRRK2, which appears not to be toxic in model systems.

## From bench to bedside: clinical trials and future prospects

Given the number of potential ways in which to interfere with LRRK2 dysfunction, it is rather early to discuss which drugs might be best developed to treat LRRK2-related PD. There are some obvious issues that may stand in the way of the assessment and development of any drug, however, and it is worth discussing those issues with the goal of identifying key experiments in the field that should be performed with a long-term view that LRRK2 is "druggable."

With the development of any drug, the first step in assessing the efficacy of the drug is to test it in a suitable animal model. To date, there are more than ten mouse models carrying different *LRRK2*-transgenes driven by different promoters (reviewed in [68]). Disappointingly, these models have not yet fully recapitulated all the clinical features seen in a PD patient. Although some DA neuronal death is observed in some of these models, the loss is not progressive and is rather limited with mild motor deficits. However, it has been reported that acute expression of mutant LRRK2 using viral vectors does replicate the DA neuronal cell loss seen in PD [51,52]. These models might be helpful in that they provide an opportunity to assess the acute application of inhibitors and might be the first level of testing novel agents with transgenic animals used for longer-term experiments, possibly using phenotypes other than DA neuronal cell loss if these can be shown to be reliable.

There are some general limitations in the development of all LRRK2 drugs. First, the potential therapeutic agent should be able to cross the blood-brain barrier (BBB). As discussed in ***Targeting LRRK2 kinase and GTPase activity***, the most promising candidate assessed biochemically and in *in vitro *models is LRRK2-IN1. When tested *in vivo*, however, it was shown that LRRK2-IN1 does not affect the phosphorylation of S910 and S935 of LRRK2 in the mouse brain, but that it does abolish phosphorylation of S910/S935 in the kidneys, indicating that LRRK2-IN1 has poor BBB permeability [62]. These data suggest that future LRRK2 inhibitors need to have distinct properties to overcome the current lack of BBB penetration.

A corollary of this issue is that poor penetration of LRRK2 therapeutic agents across the BBB could cause an inadvertent accumulation of the agents in peripheral tissues. This could raise a potentially important toxicity problem, as, for example, it was recently shown that knocking down LRRK2 in mice had deteriorating effects on kidneys [69,70]. It would therefore be important to monitor the function of organs in which LRRK2 is highly expressed, particularly with regard to kidney and lung function. LRRK2 is also highly expressed in B cells, so immunological function should be assessed.

Another major problem in the development of LRRK2 drugs is the selectivity of the therapeutic agents. Even selective LRRK2 medications could have significant off-target effects if used at high concentrations for an extended period. For example, it is well-established that no kinase inhibitor is exclusively selective to a single kinase [71]. If the concentration of the inhibitor is high enough, it could inhibit other structurally similar kinases and cause multiple side effects, a particular problem for extended use.

Assuming LRRK2 drugs were on hand, one difficult question would be when it would be most appropriate to start treatment. One the one hand, it would be logical to initiate prophylactic treatment in early adulthood in mutation carriers before the development of classic PD symptoms. In this case, appropriate therapy could potentially prevent or delay neuronal degeneration. One the other hand, mutations in the *LRRK2 *gene have incomplete penetrance and variable age at onset. Therefore, treatment of carriers could lead to long-term treatment of individuals who will not necessarily express the disease phenotype in their lifetimes, which presents both ethical and financial hurdles. This might then lead to the alternative view that any therapy should be restricted to individuals who have parkinsonism. It is also relevant that some of the motor aspects of PD are treatable, if imperfectly, by levodopa and by surgical approaches such as deep brain stimulation, so a LRRK2-based strategy would have to have proven benefit compared to current treatments. Efficacy, measurement of the progression of PD symptoms and the safety of treatments over extended periods will have to be considered in the development of LRRK2 therapeutic regimens.

Another reasonable question is whether therapeutics based on LRRK2 could also be beneficial for sporadic PD. Ultimately, this question cannot be answered without a therapeutic agent in hand, and in all likelihood this agent would be one that has proven benefit in LRRK2 cases. If one had an appropriate compound, however, it would be possible to address the hypothesis whether LRRK2 PD and sporadic PD are mechanistically linked, so it is worth thinking about the probability that this hypothesis would be supported.

On the one hand, LRRK2 is a defined subset of PD where, as we have discussed, there is even some question whether all mutations work in the same way. This would suggest that LRRK2 mechanisms would be relevant only for LRRK2 PD, perhaps even for single mutations. On the other hand, LRRK2 cases are similar clinically, and sometimes pathologically, to sporadic PD; therefore, it is possible that the underlying mechanisms are similar. In further support of the idea that LRRK2 may play a role in sporadic PD, there is a signal around the *LRRK2 *locus in GWAS in sporadic PD that is not accounted for by specific *LRRK2 *mutations [4]. Although the genetic basis of this association is unclear, one interpretation is that changes in the regulation of WT LRRK2 account for some of the lifetime risk of PD. By extrapolation, this would support the idea that LRRK2 is mechanistically linked to sporadic disease and would predict that LRRK2 therapies should be tried in idiopathic disease.

Recently, LRRK2 has been associated with an increased risk in some cancers, in particular melanoma [72,73]. PD patients with G2019S mutations have a threefold increased risk of developing melanoma before the onset of PD [72]. Whether a kinase inhibitor of LRRK2 would have any impact on cancer is very difficult to predict, but might be considered a potential extra target for drug development.

A biomarker of LRRK2 is needed to answer several of the above questions. Demonstration that a given drug has the desired effect in humans, that is, shows engagement of the target, will be crucial for deciding whether a given treatment has actually tested the underlying hypothesis. Furthermore, having a proximate biomarker for pathological LRRK2 activity would also be helpful in deciding whether the protein is involved in sporadic PD. What such a biomarker would look like is uncertain, although the action of LRRK2 kinase on a target protein is an obvious possibility. Development of these types of markers is eagerly awaited.

## Conclusion

We have discussed some therapeutic intervention points for LRRK2 PD based on what is currently known about LRRK2 function and LRRK2-induced cytotoxicity. Although LRRK2 has been implicated in many cellular pathways, including apoptosis, cytoskeleton dynamics, protein translation and other cell signaling cascades, current data are predominantly descriptive and do not explain much of the underlying molecular mechanisms. The lack of this knowledge makes the development of drugs for LRRK2-associated PD difficult. Therefore, elucidation of the exact molecular mechanisms of the neurodegenerative processes caused by different mutations in LRRK2 would be very valuable in the design of LRRK2-specific therapeutics. Of all the proven pathogenic LRRK2 mutations, only G2019S substitution has augmented phosphorylation activity. Data on other mutants do not support significant changes in their kinase function, but they do indicate that other mutants cause toxicity. This fact allows us to propose that the therapeutic strategy for PD caused by different LRRK2 mutations could be diverse and may be dependent on the unique pathogenic mechanism conferred by each substitution.

## Abbreviations

BBB: blood-brain barrier; COR: C terminus of Ras of complex protein; DA: dopaminergic; LRR: leucine-rich repeat; LRRK2: leucine-rich repeat kinase 2; PD: Parkinson's disease; ROC: Ras of complex protein.

## Competing interests

The authors declare that they have no competing interests.

## Authors' contributions

INR, RC and MRC jointly wrote the manuscript and approved of the final manuscript.

## Pre-publication history

The pre-publication history for this paper can be accessed here:

http://www.biomedcentral.com/1741-7015/10/20/prepub
